# Improving immunisation timeliness in Aboriginal children through personalised calendars

**DOI:** 10.1186/1471-2458-13-598

**Published:** 2013-06-20

**Authors:** Penelope Abbott, Robert Menzies, Joyce Davison, Louise Moore, Han Wang

**Affiliations:** 1 Aboriginal Medical Service Western Sydney, Sydney, Australia; 2 Department of General Practice, University of Western Sydney, Sydney, Australia; 3 Department of Paediatrics and Child Health, National Centre for Immunisation Research and Surveillance, University of Sydney, Sydney, Australia

**Keywords:** Immunisation, Indigenous, Aboriginal, Timeliness of immunisation, Delayed immunisation

## Abstract

**Background:**

Delayed immunisation and vaccine preventable communicable disease remains a significant health issue in Aboriginal children. Strategies to increase immunisation coverage and timeliness can be resource intensive. In a low cost initiative at the Aboriginal Medical Service Western Sydney (AMSWS) in 2008–2009, a trial of personalised calendars to prompt timely childhood immunisation was undertaken.

**Methods:**

Calendars were generated during attendances for early childhood immunisations. They were designed for display in the home and included the due date of the next immunisation, a photo of the child and Aboriginal artwork. In a retrospective cohort design, Australian Childhood Immunisation Register data from AMSWS and non-AMSWS providers were used to determine the delay in immunisation and percentage of immunisations on time in those who received a calendar compared to those who did not. Interviews were undertaken with carers and staff.

**Results:**

Data on 2142 immunisation doses given to 505 children were analysed, utilising pre-intervention (2005–2007) and intervention (2008–2009) periods and a 2 year post-intervention observation period. 113 calendars were distributed (30% of eligible immunisation attendances). Improvements in timeliness were seen at each schedule point for those children who received a calendar. The average delay in those who received a calendar at their previous visit was 0.6 months (95% CI -0.8 to 2.6) after the due date, compared to 3.3 months (95% CI −0.6 to 7.5) in those who did not. 80% of doses were on time in the group who received a calendar at the preceding immunisation, 66% were on time for those who received a calendar at an earlier point and 57% of doses were on time for those who did not receive a calendar (P<0.0001, Cochran-Armitage trend test). Interview data further supported the value and effectiveness of the calendars as both a prompt to timely immunisations and a community health education project without undue resource implications.

**Conclusions:**

Personalised calendars can increase the timeliness of immunisations in Aboriginal children. This simple, low cost tool appears practicable and effective in an Aboriginal community setting in improving early childhood vaccination timeliness and has high potential for local adaptation to suit the needs of diverse communities.

## Background

The timeliness of immunisations in Aboriginal and Torres Strait Islander children is considered the current benchmark of program effectiveness given the improvements in immunisation coverage in recent years [1]. Immunisation is considered timely when received at the earliest appropriate age, defined as within 30 days of the recommended age [2]. Delayed immunisation and illness from vaccine-preventable disease remains a significant problem in Aboriginal communities [1-3]. Although Aboriginal children have immunisation coverage levels similar to non-Indigenous children by 24 months of age [4,5], the disparity in timeliness remains, reported to be 22% for the third dose of DTP and 7vPCV, due at 6 months of age [6]. Notably, immunisation coverage is no better in the more accessible urban areas than in remote communities and in several studies has been worse [1,7,8].

Delayed immunisation, particularly in the first year of life, puts Aboriginal children at increased risk of serious morbidity from diseases that have more serious outcomes in young infants (such as pertussis) or are much more common in Aboriginal infants (such as pneumococcal diseases). There is little information on barriers to timely immunisation specific to the Australian Aboriginal population. Caregiver disagreement with immunisation appears to be less common for Aboriginal Australians [9]. Other factors which may contribute to decreased vaccination timeliness in Aboriginal children have been identified in international studies. Socioeconomic disadvantage is the strongest predictor of under-immunisation [10-12] and Aboriginal Australians continue to experience marked disadvantage [13]. In a New Zealand study, predictors of lower vaccination coverage and timeliness within primary care practices were socio-economic deprivation, practice populations with a higher proportion of Maori and staff shortages [14]. Of further potential relevance, incomplete or delayed immunisation has been associated with large family size [15], residential mobility [16], poor caregiver knowledge of the immunisation schedule [17] and a lack of parental concern about immunisation timing [18].

Community based interventions to improve immunisation rates may do so by (a) increasing community demand for immunisation, such as through education and patient reminders, (b) improving access to immunisation services and (c) improving provider systems, such as through decreasing missed immunisation opportunities [17,19,20]. Effectiveness is increased when interventions are implemented in combination [19]. In harder to reach populations where children face the most barriers to timely immunisation, standard strategies may be less effective [21], however more intensive systems such as manual outreach, tracking and home visits are costly and labour intensive [19].

Despite the longstanding recognition that delayed immunisations and increased vaccine preventable disease in Aboriginal children are a significant public health problem in Australia, there is a paucity of information on strategies which may be effective in promoting timely immunisation without placing undue burden on already overworked services such as Aboriginal community controlled health organisations. This paper reports on a simple, low cost activity designed to promote timely childhood immunisation through the use of personalised calendars, which was undertaken at the Aboriginal Medical Service Western Sydney (AMSWS) in 2008–2009. The Calendar Project was informed by a program which had been successful in improving immunisation timeliness in African-American children attending public health centres in Missouri [22,23].

## Methods

### Study setting

The AMSWS is a large Aboriginal community controlled health service which provides multidisciplinary primary health care to the Aboriginal community of western Sydney. The majority of AMSWS clients come from the suburbs adjoining the service, which are amongst the most socioeconomically disadvantaged in Australia [24]. Some clients attend from the wider area of western Sydney or during visits to the area, including from rural communities throughout New South Wales. A number of AMSWS clients also attend other Aboriginal and non-Aboriginal primary care services for their health care needs, which means multiple health care providers can be involved in the delivery of the scheduled childhood immunisations.

At the time of this study, immunisations were given by practice nurses and Aboriginal health workers (AHWs) after a child had been seen by a general practitioner (GP). Clients attended most GP consultations on a drop-in basis, all consultation costs were covered under universal health insurance and transport to the clinic was available for carers attending the AMSWS with young children.

### Intervention

The project was approved by the Board of the Aboriginal Medical Service Western Sydney and formal consent for participation in the interviews was obtained. Ethics approval was received from the Aboriginal Health and Medical Research Council Ethics Committee.

The intervention period for the Calendar Project ran from January 2008 – September 2009. The target group were children attending the AMSWS for any immunisation at the scheduled dose points of 2, 4, 6, 12 and 18 months of age. A community survey prior to commencement informed the project. The main consumer recommendation was that the vaccine preventable diseases should be noted on the calendar as carers were not always clear on the reason behind immunisation recommendations.

**Figure 1:**
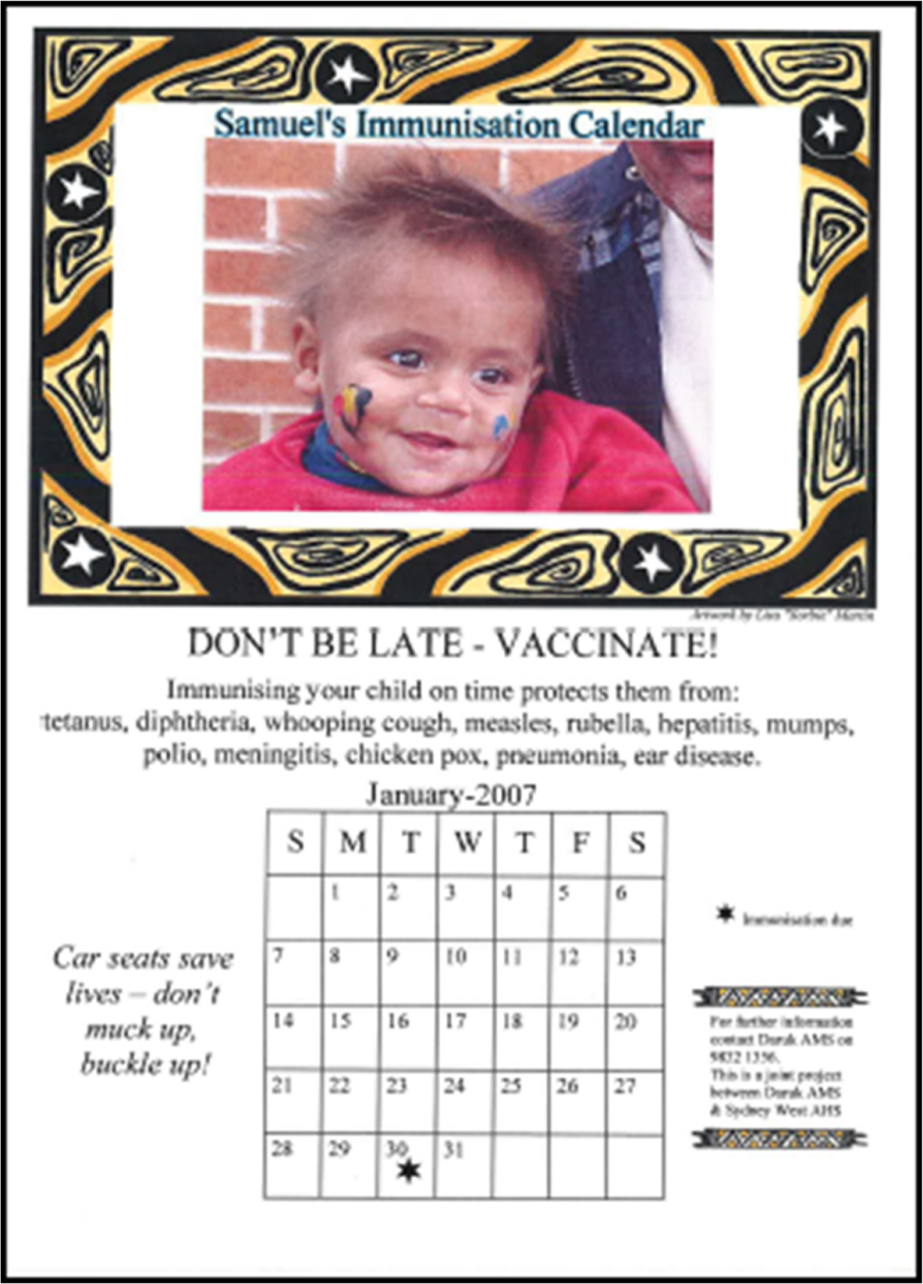
Example of personalised immunisation calendar.

The calendar was an A4 sized laminated page showing the month and day when the child’s next immunisation was due and a picture of the child (Figure 1). The slogan “Don’t be late – vaccinate” featured prominently. Aboriginal artwork, the list of diseases targeted in the immunisation schedule and short health promotion messages were included. The artwork and health promotion messages varied according to the age of the child. The child’s photo was generated either before or after the immunisation by webcam into a Microsoft Access computer program, which required entry only of the patient’s name, age category and the date the next immunisation was due. No data were stored by the program. The calendar was usually generated by the AHW project staff.

GPs, AHWs or nurses were able to offer calendars to carers at any childhood immunisation. Calendars could also be requested by carers and there were signs in the waiting room advertising the program. The usual immunisation process and medical care were unchanged. Pragmatic service level factors, caused by staff shortages and competing demands on clinic space, largely determined whether a client was offered a calendar or could receive one when they requested it. In particular, as the program was stored on one computer in a shared clinic room, the calendar could not be generated if that room was not available at the time.

### Analysis

In a retrospective cohort design, data from the Australian Childhood Immunisation Register (ACIR) were collected for all recorded immunisations of children who had ever had any immunisation at the scheduled dosing points of 2, 4, 6, 12 and 18 months at the AMSWS between January 2005 and September 2011. The data included immunisations given by non-AMSWS providers. To increase the completeness and accuracy of the data, review of the immunisation records in the AMSWS clinical databases (Ferret, Pen Computer Systems, and Medical Director, Health Communication Network), supplemented by hand checking of paper-based medical records when discrepancies were noted, was also done for all children.

The timeliness of immunisations of children who received at least 1 calendar during the 21 month study period of January 2008 to September 2009 was compared to those who did not receive a calendar. The timeliness of immunisations of children from the pre-study period was also examined.

Two indicators of timeliness were calculated: the mean vaccination delay (date of vaccination minus due date), and the proportion of children who were vaccinated <31 days after the due date. Indicators were compared in children who had received at least 1 calendar during the 21 month study period, those who did not receive a calendar over the same period, and children from the pre-study period. For the ‘mean delay’ analysis, the mean and 95% confidence interval (CI) were calculated from the vaccination delays of individual children. Statistical significance of the difference between means was assessed by whether or not CIs overlapped. The ‘no calendar’ group included children who had never received a calendar as well as children who had received a calendar previously to remind them about a previous dose, but not the current dose. Therefore this analysis assessed only the effectiveness of the calendar on the immediately subsequent immunisation. In order to reduce the influence of a small number of outlier doses that were very delayed, the analysis was repeated excluding doses that were ≥23 months late for 4 and 6 month schedule points and ≥19 months late for 12 and 18 month schedule points. The proportion vaccinated on time was included as a second indicator to reduce the impact of outliers but without excluding any data and to assess the impact of the calendar on subsequent immunisations, not just those doses that were specifically prompted by the calendar. For the ‘proportion on time’ analysis, children who had never received a calendar were analysed separately to those who had received a calendar to remind them about a previous dose, but not the current dose. Trends in the proportion of children vaccinated on time were tested for statistical significance using the Cochran-Armitage trend test [25]. Analysis was conducted in SAS version 9.3 [26].

To enhance understanding of the quantitative data, a qualitative evaluation was also undertaken, consistent with summative evaluation [27]. Carer and AMSWS staff views of the calendar project were collected in semi-structured interviews lasting between 10–20 minutes. A total of 14 carer and 11 staff members (4 Aboriginal health workers, 3 registered nurses and 4 GPs) were interviewed as to their perceptions of the calendar and its usefulness and barriers to its use. Interviews were audio-taped, transcribed and then analysed thematically to enhance understanding of the effectiveness, feasibility and potential generalisability of the calendar project as a strategy for promoting immunisation timeliness in the Aboriginal Medical Service setting.

## Results

Data on 2142 immunisation doses given to 505 children were analysed, comparing the pre-intervention (2005–2007) and intervention period (January 2008 - September 2009), with a subsequent observation period from October 2009- September 2011. During the intervention period, 113 calendars were given at 377 (30%) eligible attendances for the scheduled 2, 4, 6 and 12 month immunisation dose points. Some children received calendars at more than 1 dose point. Of the total 189 children who attended for immunisation during the intervention period, 79 children (42%) received at least one calendar. Calendars given at the 18 months vaccination point were not included in the analysis as the timeliness of the 4 year dose point was not assessed. Also not included in the analysis are 6 children who received calendars but had no record of subsequent immunisations.

The mean immunisation delay for all children who received at least one immunisation at the AMSWS between 2005 and 2011 is shown in Table 1. The delay at the 2, 4, 6, 12 and 18 month scheduled dose points for immunisations given by both AMSWS and non-AMSWS providers is presented. Post-intervention data collection continued for 2 years to allow sufficient time for inclusion of delayed immunisation data. This data also includes doses given to children born after the intervention period, forming part of the comparison group.

**Table 1:** Effectiveness of calendar – delay in subsequent immunisations (outliers included)

**Study groups**	**Year of immunisation**	Type of immunisation *	**Calendar received at last immun-isation**	**No. of doses**			**Mean delay (months)**			95% CI **	
				**Given at AMSWS**			**Given at AMSWS**				
				**No**	**Yes**	**Total**	**No**	**Yes**	**Total**	**Total**	
Pre-Calendar program	2005-2007	02 M		86	176	262	1.0	0.8	0.9	−2.2	3.9
		04 M		97	163	260	1.3	2.2	1.9	−1.6	5.4
		06 M		79	167	246	2.0	3.3	2.9	−0.5	6.2
		12 M		68	162	230	2.2	2.7	2.5	0.0	5.1
		18 M		53	91	144	1.4	3.5	2.7	0.1	5.3
		all		383	759	1142	1.6	2.4	2.1	−0.9	5.0
Calendar Program -intervention period Jan 2008-Sept 2009 - observation period Oct 2009-Sept 2011	2008-2011	02 M	N/A	47	111	158	0.6	0.5	0.5	−1.6	2.7
		04 M	No	47	88	135	2.4	3.8	3.3	−2.0	8.7
			**Yes**	**1**	**31**	**32**	**2.5**	**0.3**	**0.4**	**−0.3**	**1.1**
		06 M	No	63	100	163	5.1	4.7	4.8	−0.6	10.3
			**Yes**	**2**	**25**	**27**	**12.8**	**0.5**	**1.4**	**−1.9**	**4.7**
		12 M	No	72	129	201	3.5	3.2	3.3	0.1	6.4
			**Yes**	**5**	**24**	**29**	**1.6**	**0.9**	**1.0**	**−0.1**	**2.1**
		18 M	No	97	139	236	5.0	4.3	4.6	1.0	8.2
			**Yes**	**3**	**16**	**19**	**0.3**	**0.9**	**0.8**	**−0.3**	**1.9**
		all	No	326	567	893	3.7	3.3	3.4	−0.6	7.5
			**Yes**	**11**	**96**	**107**	**3.4**	**0.6**	**0.9**	**−0.8**	**2.6**

* Immunisations defined as to the age of the child in months when the vaccine was due according to the NSW Immunisation schedule. The specific vaccines which were used in the analysis to assess immunisation status were: 02M= dTpa1; 04M= dTpa2; 06M=dTpa3; 12M= MMR; 18M=varicella.

** CI very large for group of children who did not receive calendars due to several outliers with very delayed vaccination.

For immunisations given at the AMSWS in the pre-intervention period the average delay was 2.4 months (95% confidence interval −0.9 to 5.0). In the intervention period the average delay was 3.3 (−0.6 to 7.5) months for doses where a calendar was not given at the child’s previous due dose, and 0.6 (−0.8 to 2.6) months where a calendar had been given at the last visit. Smaller average delays were seen at every schedule point for doses following receipt of a calendar compared to doses not following receipt of a calendar, although no differences were statistically significant, as seen by overlapping 95% confidence intervals. While this pattern was present for doses given at AMSWS, it was not as evident for doses given by other providers. For doses given by other providers there was little difference in delay following calendar receipt (3.4 months) and not following calendar receipt (3.7 months), although the significance of this is unclear given that the number who had received a calendar was small (n=11).

When the 35 very delayed outlier doses from the calendar and post-calendar periods were excluded (1 received a calendar and 34 did not), the average delays at all dose points became statistically significantly different. Including data from all providers, when no calendar had been given at the last visit, the total delay was 2.2 (1.9 to 2.5) months, and 0.7 (0.4 to 1.0) months when a calendar had been given at the last visit.

The percentage of all vaccinations during the intervention period that were given on time (<31 days after due date) in those receiving immunisations at the AMSWS was lowest amongst those who had never received a calendar (57.2%), followed by those who had received a calendar previously to remind them about a previous dose but not the current dose (66.4%) and highest for those who had received a calendar at their previous visit to remind them of the current dose (80.4%) (Table 2). This trend was highly significant (P<0.0001), and also significant at each schedule point (Table 2). The trend was less consistent for the small number of doses given by non-AMSWS providers.

**Table 2:** Effectiveness of calendar – trend percentage of doses on time

**Type of immunisation (scheduled immunisation point)**	**Calendar received**	**Percentage of doses on time (delay <31 days)**			**Cochran-Armitage trend test**
		**Immunisation given at AMSWS**			
		**No**	**Yes**	**Total**	**p value**
02 M	Never	83.0%	89.2%	87.3%	N/A
04 M	Never	68.1%	56.8%	60.7%	N/A
	Last visit *	**0.0%**	**90.3%**	**87.5%**	N/A
06 M	Never	40.0%	43.2%	41.8%	0.0012
	Before last visit**	**66.7%**	**57.9%**	**59.1%**	
	**Last visit**	**0.0%**	**80.0%**	**74.1%**	
12 M	Never	49.3%	55.0%	52.7%	0.0106
	**Before last visit**	**40.0%**	**79.3%**	**73.5%**	
	**Last visit**	**40.0%**	**79.2%**	**72.4%**	
18 M	Never	42.5%	46.3%	44.7%	<.0001
	**Before last visit**	**58.8%**	**67.7%**	**64.6%**	
	**Last visit**	**100.0%**	**87.5%**	**89.5%**	
all types	Never	53.8%	59.2%	57.2%	<.0001
	**Before last visit**	**56.0%**	**69.6%**	**66.4%**	
	**Last visit**	**45.5%**	**84.4%**	**80.4%**	

* Last visit = calendar was given at immediately preceding scheduled immunisation point, prompting this particular immunisation.

** Before last visit= calendar had been given at a previous immunisation point as a reminder for a previous dose, but not at the immunisation immediately preceding this scheduled immunisation.

N/A = Trend test not applicable.

### Interviews with carers and staff

The carers interviewed believed strongly that the calendar helped to remind them to have the next immunisation on time. The calendars were usually displayed prominently in the home. The most valued features of the calendar were the child’s photo and the Aboriginal art work. Several participants stated the calendar made them feel special and that projects like this were one of the reasons they chose to attend the AMSWS where their Aboriginality was celebrated.

“It was all laminated, so you can keep it for when they got older as well. And the photo’s on there, and it’s got all our artwork on there, the Koori designs and borders and that, so that’s really good. Because it’s something just for us.”

Staff members believed the project had increased the awareness of staff and carers of the importance of timely childhood immunisations and prompted them to discuss timeliness and catch up schedules with carers. One AHW reported it helped her to understand better how to determine when the next immunisation was due, however another believed she still found it difficult to determine when an immunisation was due if the child was behind schedule and there was a risk she had sometimes put the wrong due date on the calendar if that had not been very clearly communicated to her.

A strong theme in the staff interviews was that the calendar was something special to offer carers which enhanced the clinical interaction.

“The mums like to have a photo taken of their babies. I think it makes the mothers feel that they and their kids are important, something special that’s been done, some attention. It takes away from it just being an immunisation, to being a bit of a social interaction.”

It was uncommon for carers to request calendars despite the Calendar Project being advertised in the waiting room. Staff reported that the only people who requested a calendar were those who had received one at a previous immunisation. Some carers reported they felt unable to ask for the calendar if it was not offered to them and were disappointed if they had not received it at an immunisation visit.

Barriers to offering the calendar were identified in the staff interviews. It was difficult to organise the calendar when the clinic was busy due to high client load or understaffing. Space shortages meant that there were both regular and unpredictable periods throughout most weeks when the computer used to generate the calendar could not be accessed as the room was unavailable. Technological issues arose periodically. If trained staff were not immediately available at the time the child presented for immunisations, calendars were not offered. Some staff did not usually refer carers for calendars even when it would have been possible to do so, and stated their reasons were uncertainty as to the value of the program and competing priorities which led them to forget. Staff reported that most carers accepted a calendar when offered unless they had been waiting a long time to see a GP. Carers often requested several copies to give to family members.

## Discussion

This study provides evidence of the effectiveness of a simple and low cost intervention in increasing the timeliness of childhood immunisations in an Aboriginal community setting. Based on a more resource-intensive program delivered in a randomised controlled trial design in the United States [22,23], this study has shown that the calendar is a tool which can be simplified, culturally adapted and delivered in an Aboriginal community controlled health organisation with low impact on usual immunisation procedures and general service delivery. The cost and staff resources required to improve immunisation timeliness in hard to reach populations can be burdensome on primary care services which are often already understaffed, and simple strategies to promote timely immunisation in these populations are greatly needed. Furthermore, health promotion activities such as the Calendar Project can be important within Aboriginal community controlled health organisations as an expression of cultural identity [28]. Staff and carers felt the calendar enhanced the clinical immunisation interaction.

The photo of the child and the Aboriginal artwork were identified as pivotal to the effectiveness of the calendars, encouraging recipients to keep and to display the calendar in their home. The calendar was therefore seen by extended family members and friends when displayed in their house and some had given copies to family members as gifts, so it may have had a health promotion impact beyond the immediate family.

Evidence relevant to the reasons behind poor timeliness in Australian Indigenous people supports the importance of socioeconomic factors and knowledge of the importance of timeliness. The calendars were most effective as a prompt for the next immunisation, but also had significant effect on increasing the timeliness of subsequent immunisations, even if no calendar was given prompting that scheduled dose. This provides evidence that, as well as providing a simple reminder of the date of the next immunisation, the calendars had an additional educational effect which affected subsequent immunisations. Both carers and AMSWS staff may have become more aware that immunisations should be given on time. It is possible that as well as increasing community demand for immunisations, the calendar project may have improved the AMSWS’s systems as an immunisation provider, such as through decreasing missed opportunities for immunisation and more clearly communicating catch up schedules to carers.

Interpretation was complicated by the presence of a small number of doses which were very delayed - 35 out of 1000 in the calendar and post-calendar periods were given ≥19 or ≥24 months after the due date. Outliers may distort statistical comparisons, particularly means [29]. Therefore, in this study we provided comparisons of means with and without the outliers. The exclusion of outliers reduced the point estimates of delay, but also reduced the 95% CI, for both calendar and non-calendar groups, so that the CIs no longer overlapped. An alternative analysis less prone to distortion by outliers, namely percentage of doses given on time, showed a clear and significant association between decreasing delay and receipt of a calendar.

The major limitation of this study was that there was no matched control group. Calendars were given out opportunistically and therefore some carers were not offered a calendar, furthermore, some declined calendars. Clinic level factors such as staff shortages, clinic waiting times and intermittent access to the equipment required were reported to be the major influences on the likelihood of being given a calendar, however staff decisions on whether to offer a calendar and carer characteristics may have affected the timeliness of immunisations in this study.

## Conclusion

This study provides evidence of the effectiveness of a simple and low cost intervention in increasing the timeliness of immunisations in the first 2 years of childhood in an urban Aboriginal community setting. Personalised calendars may be an effective strategy to increase the timeliness of childhood immunisations and decrease the burden of vaccine-preventable disease. The usefulness of this tool was increased by the minimal impact on service delivery. There is potential for cultural adaptation of the calendars to meet local community needs, including in other Aboriginal communities and in diverse communities in which timeliness of immunisation is a problem.

### Consent

Written informed consent was obtained from the patient’s guardian/parent/next of kin for the publication of this report and any accompanying images.

## Competing interests

The authors declare that they have no competing interests.

## Authors’ contributions

The study was designed by PA, RM, JD and LM and implemented by PA, JD and LM. Data analysis was undertaken by PA, RM and HW. The manuscript was drafted by PA and RM and all authors contributed to and approved the final manuscript.

## Pre-publication history

The pre-publication history for this paper can be accessed here:

http://www.biomedcentral.com/1471-2458/13/598/prepub
